# Platelet‐Rich Fibrin Mediates Beneficial Effects on Adipose‐Derived Stem Cells via Increased Levels of Key Cytokines

**DOI:** 10.1111/wrr.70040

**Published:** 2025-05-21

**Authors:** Nikolaus Wachtel, Luisa Weber, Nicholas Moellhoff, Constanze Kuhlmann, Riccardo E. Giunta, Paolo Alberton, Denis Ehrl, Severin Wiggenhauser

**Affiliations:** ^1^ Division of Hand, Plastic and Aesthetic Surgery University Hospital, LMU Munich Germany; ^2^ Department of Plastic, Reconstructive and Hand Surgery, Burn Center for Severe Burn Injuries Klinikum Nuremberg Hospital, Paracelsus Medical University Germany

**Keywords:** adipose‐derived‐stem cells, platelet‐low‐plasma, platelet‐rich‐fibrin, TGF‐beta1

## Abstract

Recent studies showcased the regenerative potential of Platelet‐Rich Fibrin (PRF) combined with Adipose‐Derived Stem Cells (ASC). PRF enhances cellular proliferation through sustained growth factor secretion which are continuously released to surrounding cells. However, its regulatory mechanisms remain unclear. ASC were isolated from liposuction and abdominoplasty samples of healthy donors, characterised via flow‐cytometry and cultured for 7 days. Four cell culture conditions were tested: (1) 10% PRF extract (PRFe), (2) 10% Platelet‐Low Plasma (PLP), (3) 10% Foetal Bovine Serum (FBS) and (4) basal medium as control. Cell viability and proliferation were assessed using AlamarBlue and PicoGreen assays, as well as live‐dead staining. Enzyme‐Linked Immunosorbent Assays quantified growth factor concentrations, while multiplex qPCR and immunocytochemical staining analysed gene and protein expression on days 1 and 7. PRFe‐supplemented cultures showed the highest viability and proliferation, significantly surpassing other groups at day 7 (*p* < 0.05). Supernatant analysis revealed significantly elevated TGF‐β1 and PDGF‐AA/BB levels in PRFe cultures at day 7 (*p* of at least < 0.05). Multiplex qPCR indicated increased expression of proliferation and pluripotency markers (NANOG, JUN, SOX2, RPS6KA4; *p* < 0.05) and fibrillar collagen (COL1A; *p* < 0.05) in the PRFe group. These findings demonstrate that PRFe significantly enhances ASC proliferation and regenerative potential. Elevated levels of TGF‐1, PDGF‐AA/BB and to a lesser extend VEGF in PRFe cultures suggest that its benefits in regenerative medicine may be linked to these cytokines' upregulation. These results underscore PRFe's potential as a key supplement for optimising ASC‐based therapies in tissue regeneration.

**Abbreviations**
ASC: adipose‐derived stem cell
BSA: bovine serum albumin
COL1A1: collagen type 1 alpha 1 chain
DAPI: 4′‐6′‐diamidino‐2‐phenylindole
DMEM: Dulbecco's modified eagle medium
ECM: extracellular matrix
EDTA: ethylene‐diaminetetraacetic acid
ELISA: Enzyme‐Linked‐Immunosorbent‐Assay
FAK‐Pathway: focal‐adhesion‐kinase‐pathway
FBS: foetal bovine serum
FDA: fluorescein diacetate
GAPDH: glyceraldehyde‐3‐phosphate dehydrogenase
JUN: Jun proto‐oncogene, ATP1‐transcription factor subunit
MSC: mesenchymal stem cells
NANOG: homebox protein NANOG isoform 1
PBS: phosphate‐buffered saline
PDGF‐AB: platelet‐derived‐growth factor AB
PDGF‐BB: platelet‐derived‐growth factor BB
PI: proprium iodide
PLP: platelet‐low‐plasma
PRF: platelet‐rich‐fibrin
PRFe: platelet‐rich‐fibrin‐extract
PRP: platelet‐rich‐plasma
PTGS2/ COX2: prostaglandin‐endoperoxide synthase 2/mitochondrially encoded cytochrome c oxidase II
PXN: Paxillin
Ras/MAPK‐pathway: Ras‐mitogen‐activated protein kinase‐pathway
RPS6KA4: ribosomal protein S6 kinase A4
SOX2: SRY‐box transcription factor 2
TGF‐β1: transforming‐growth‐factor‐β1
VEGF: vascular‐endothelial growth factor

## 1 Introduction

Platelet‐Rich Fibrin (PRF) is a second‐generation platelet‐rich‐plasma obtained by centrifugation of autologous blood without the addition of anticoagulants [1]. Due to its easy manufacturing and good availability, PRF has been successfully used in several fields of regenerative clinical medicine, including oral, maxillofacial and plastic surgery, dermatology, sports medicine and dental medicine [2, 3, 4, 5]. A significant advantage of its precursor, Platelet‐Rich Plasma (PRP), is the shorter preparation time and no need to add anticoagulants during the manufacturing process [6, 7]. Indeed, a main reason for the development of PRF was to prevent hypersensitivity reactions that can be caused by bovine anticoagulants necessary for the production of PRP [1, 8]. Additionally, a major limitation of PRP is the rapid release of ~95% of growth factors shortly after activation with thrombin. Conversely, the polymerised fibrin structure in PRF allows for a stable secretion of growth factors and other cytokines for up to 7 days [9, 10, 11]. These include Platelet‐Derived Growth Factor AB and BB (PDGF‐AB and BB), Vascular Endothelial Growth Factor (VEGF) and Transforming Growth Factor‐β1 (TGF‐β1) and are believed to play a role in promoting angiogenesis, cell migration and tissue regeneration [3, 12].

Each cytokine acts through distinct molecular signalling pathways, for example, the Ras‐mitogen‐activated protein kinase‐pathway (Ras/MAPK‐pathway) and focal‐adhesion‐kinase (FAK)‐pathway, providing multiple potential research angles to understand the underlying mechanisms. Moreover, fibrin itself is a key element in haemostasis as well as one of the most abundant proteins in extracellular matrix (ECM), hereby providing a stable matrix for subsequent tissue regeneration [13, 14]. The liquid extract released from the fibrin scaffold over time contains all the biologically active components and provides a soluble form of PRF that can be administered through injection, known as Platelet‐Rich‐Fibrin‐extract (PRFe) [15]. Fibrin as well as PRFe are believed to be responsible for the positive properties of PRF in regenerative medicine [16, 17, 18].

Another emerging technique in the field of regenerative medicine is the transplantation of tissue engineered Adipose‐Derived‐Stem cells (ASC) to allow for wound healing and/or defect reconstruction. Stem cells are generally classified into two primary categories: pluripotent embryonic stem cells, derived from the inner cell mass of the blastocyst and adult (somatic) stem cells, which possess self‐renewal capacity and are typically restricted to differentiating into cell types within their tissue of origin [19]. ASC represent a subset of mesenchymal stem cells (MSC), predominantly isolated from adipose tissue, though MSC are also present in other mesenchymal tissues such as bone marrow and the umbilical cord. These mesenchymal progenitor cells are characterised by their ability to adhere to plastic surfaces, their fibroblast‐like morphology and their multipotency, which enables differentiation into various mesenchymal lineages, including adipocytes, osteoblasts and chondrocytes [20].

Especially ASCs have attracted considerable interest in the field of regenerative medicine due to their ready availability, robust proliferative capacity and differentiation potential [21, 22, 23].

Both stem cells and autologous platelet concentrates have been extensively assessed for their respective potential in regenerative medicine in the previous decades [9, 22]. Interestingly, only little research has gone into merging the beneficial effects of PRF and ASC in wound healing. Moreover, the majority of studies only assess possible beneficial effects of PRP, which is likely to have inferior properties when compared to PRF [6, 7, 24]. This study therefore aims to assess whether PRFe, a derivative of PRF, can exert a beneficial effect on ASCs, possibly creating a more potent and efficient approach for treating wound healing and tissue regeneration by leveraging and merging the advantageous characteristics of both techniques. For this, we assessed possible beneficial effects of PRFe on various markers of cell viability and tissue regeneration of ASC in vitro. The parameters were evaluated at three time points—on the day of preparation, at day 3, and day 7 to evaluate the effect of short, medium, and long‐term exposure of PRFe on ASCs.

## 2 Methods and Materials

### 2.1 Cell Isolation

Human ASCs were harvested from fat tissue obtained by liposuction and abdominoplasties performed at the Department of the authors. Written consent was given by all patients prior to the operation as part of the study's approval by the institutional ethics committee (registration number: 21‐0138). Fat tissue sourced from abdominoplasties was minced and placed in a 50 mL tube. We used a modified protocol described by Bunnell et al. [25]. A 0.15% collagenase II‐solution (Worthington Biochemical Corp., Lakewood, NJ, USA) in phosphate buffer saline (PBS) (Gibco, Thermo Fisher Scientific, Waltham, MA, USA) was added at a 2:1 ratio to enzymatically digest the tissue. After a 35 min incubation period at 37°C and periodic shaking, the enzymatic reaction was stopped by adding cell culture medium containing foetal bovine serum (FBS) (Gibco, Thermo Fisher Scientific, Waltham, MA, USA). Centrifugation at 1200 g for 10 min led to the formation of three phases including the stromal‐vascular‐fraction on the bottom, which was used to further isolate the stem cells. The latter was filtered through a 100 μm nylon cell strainer (Falcon), transferred to another tube and subjected to another round of centrifugation at 300 g for 5 min. The cell pellet forming at the bottom of the tube contains the ASC and was resuspended in fresh cell culture medium in a T175 flask (Thermo Fisher Scientific, Waltham, MA, USA) for culture expansion. Cell culture was maintained using Dulbecco's Modified Eagle Medium (DMEM) (Gibco, Thermo Fisher Scientific, Waltham, MA, USA) consisting of 1% (100 μg/mL) Penicillin/Streptomycin (Sigma‐Aldrich, Merck Technology, St. Louis, MO, USA), 1% (100 μg/mL) Amphotericin B (Sigma‐Aldrich, Merck Technology, St. Louis, MO, USA) and 10% FBS (Gibco, Thermo Fisher Scientific, Waltham, MA, USA) at 37°C and 5% CO_2_ in a humidified atmosphere until reaching 80% confluency. Cells were then either cryopreserved in freezing medium or seeded for further experiments.

### 2.2 Cell Culture

ASC were seeded in biological triplicates on a 12 well‐plate at a density of 3 × 10^4^ cells per well in standard cell culture medium +10% FBS. After adhesion, the medium was discarded, the cells were washed with PBS once and the medium was then replaced with the treatment medium. The following four groups were established: (1) standard cell culture medium +10% Platelet‐Rich‐Fibrin extract (PRFe) (2) standard cell culture medium +10% Platelet‐Low‐Plasma (3) standard cell culture medium +10% FBS and (4) standard cell culture medium without the addition of any growth supplement (basal medium).

### 2.3 Flow‐Cytometry

According to a joint statement of the international Federation for Adipose Therapeutics and Science (IFATS) and the international Society for Cellular Therapy (ISCT) the following surface markers were used to immunophenotypically characterise the isolated cells at passage 0: CD44, CD29, CD13, CD73, CD90, CD105, CD31, CD45, CD235 (all BioLegend) [26].

Each cell line was seeded in a T75 Flask (Thermo Fisher Scientific, Waltham, MA, USA) until reaching 90% confluency (*n* = 3). To obtain a single cell solution, cells were trypsinized and maintained in flow‐cytometry buffer consisting of 1 mM Ethylenediaminetetraacetic acid (EDTA) (PanReacAppliChem, Darmstadt, Deutschland), 1% bovine serum albumin (BSA) (Gibco, Thermo Fisher Scientific, Waltham, MA, USA) and PBS. 1 μL per antibody was then applied to each sample, incubating for 30 min at +4°C.

Control samples included unstained controls and appropriate isotype stain controls.

Finally, samples were run on a BD LSRFortessa Flow Cytometer and the data were analyzed using FlowJo V10.10.0 (BD BioSciences). (Table 1).

**TABLE 1:** Antibodies for flow‐cytometry.

Surface marker	Isotype	Conjugate	Manufacturer
CD44	Mouse IgG1, k	Pacific Blue	BioLegend, Biotechnology, San Diego, CA, USA
CD29	Mouse IgG1	FITC	eBioScience, San Diego, CA, USA
CD13	Mouse IgG1	BV711	BioLegend, Biotechnology, San Diego, CA, USA
CD73	Rat IgG1	AF700	BioLegend, Biotechnology, San Diego, CA, USA
CD90	Mouse IgG1	BV786	BioLegend, Biotechnology, San Diego, CA, USA
CD105	Mouse IgG1	APC	BioLegend, Biotechnology, San Diego, CA, USA
CD31	Mouse IgG1	PE‐Cy7	BioLegend, Biotechnology, San Diego, CA, USA
CD45	Mouse IgG1	PerCP	BioLegend, Biotechnology, San Diego, CA, USA
CD235a	Mouse IgG2a	PE	BioLegend, Biotechnology, San Diego, CA, USA

### 2.4 Platelet‐Rich‐Fibrin‐Extract and Platelet‐Low‐Plasma

**FIGURE 1:**
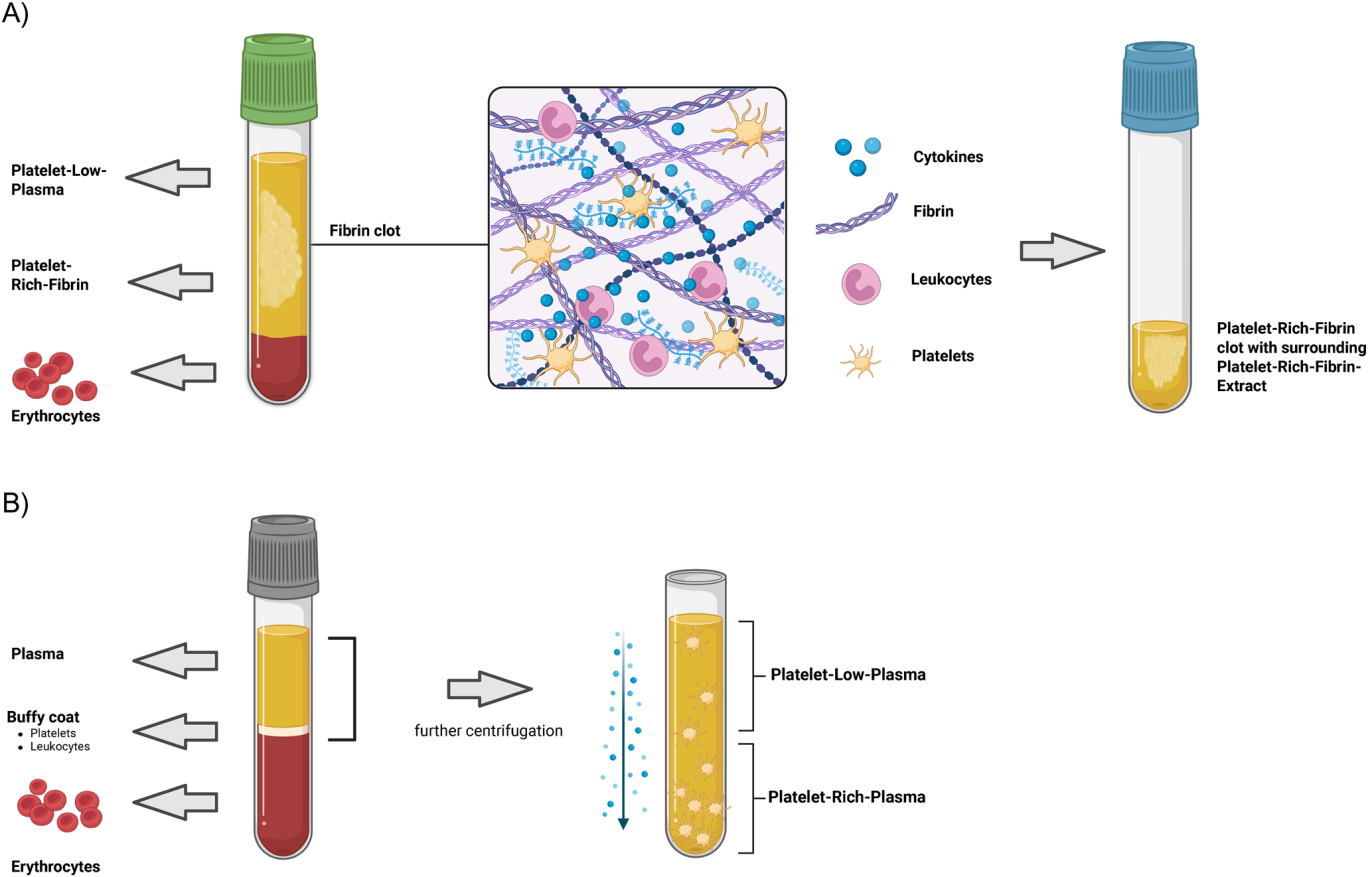
(A) Platelet‐Rich‐Fibrin after centrifugation of venous blood without anticoagulants: The erythrocytes in the lower layer, the platelet‐rich fibrin clot in the middle layer, and the platelet‐poor plasma in the upper layer are visible. Within the fibrin clot, the activated platelets and the fibrin build a three‐dimensional matrix that incorporates various cytokines, such as PDGF‐AB, PDGF‐BB, VEGF and TGF‐beta1. The formed PRF is transferred into another tube, where the released extract forms a yellow fluid, the PRFe. (B) After centrifuging 3.2% citrate blood samples, a three‐layer formation is observed. A second centrifugation of the upper plasma leads to the separation of platelet‐low plasma and platelet‐rich‐plasma. Created with BioRender.com.

Healthy volunteers provided written consent before two vials (10 mL each) of peripheral vein blood were drawn (*n* = 5). The whole blood samples were centrifuged for 14 min at 1500 rpm without or with (for Platelet‐Low‐Plasma) the addition of anticoagulants (Sarstedt AG & Co. KG Nuermbrecht, Germany) and rested for an additional 5 min at the bench top [27]. The fibrin polymerised and formed a gel‐like substance, residing at the top of the blood collection tube. To separate the erythrocytes at the bottom, the PRF was lifted using forceps (Fuhrmann GmbH, Much, Germany) and cut off 2 mm above the pellet interface. The PRF was then transferred to a 15 mL collection tube and remained at room temperature for 30 min, during which the fibrin clot started to release an initial extract (PRFe). Each vial of blood yielded approximately 1 mL of PRFe and was stored at 4°C until further usage (Figure 1). For cell culture application, the PRFe from these five individual donors was combined and utilised as a pooled supplement.

In contrast to the preparation of PRF, 2 vials (10 mL each) whole blood samples were drawn using blood collection tubes that contained 0.106 mol/L (3.2%) citrate (Sarstedt AG & Co. KG Nuermbrecht, Germany) and centrifuged at 3000 rpm for 10 min [28]. Platelet‐Low‐Plasma (PLP) was then transferred to a different tube and subjected to an additional round of centrifugation. The PLP was then collected and stored at 4°C until further use. Similar to the PRFe, we used a pooled supplement as our cell culture growth supplement.

### 2.5 Cell‐Viability‐Assay

According to the manufacturer's in, struction, the cell Culture medium in each group (*n* = 3) was removed and replaced with a 10% AlamarBlue Cell Viability Reagent solution (Invitrogen, Thermo Fisher Scientific, Waltham, MA, USA) diluted in phenol‐red‐free DMEM (Gibco, Thermo Fisher Scientific, Waltham, MA, USA) at days 1, 3 and 7 of culture. After 2 h of incubation, 100 μL of the supernatant from each well was transferred to a black 96‐well plate (Thermo Fisher Scientific, Waltham, MA, USA) and absorption intensity was measured in triplicates at 560 nm (extinction) and 590 nm (emission) in a plate reader (Infinite M Plex, TECAN, Männerdorf, Switzerland). A blank control group was subtracted from each measurement to correct for background signal.

### 2.6 DNA‐Quantification‐Assay

Using the protocol provided by the manufacturer, the cell culture medium was discarded and the cells were rinsed with cold PBS. Plated cells were lysed using RLT Buffer (Qiagen, Hilden, Germany) for 10 min on an IKA shaker, and the resulting lysates were collected in 1.5 mL LoBind Tubes (Eppendorf, Hamburg, Germany). 20 μL Proteinase K (PanReacAppliChem, Darmstadt, Deutschland) was added to each tube, followed by another incubation of 10 min at 56°C. We used the DNeasy Blood & Tissue Kit (Qiagen, Hilden, Germany) to extract and purify DNA from the samples, which was pipetted into a black 96 well plate (Thermo Fisher Scientific, Waltham, MA, USA) in 100 μL duplicates (*n* = 3). The PicoGreen reagent from Quant‐iT PicoGreen dsDNA Assay Kit (Invitrogen, Thermo Fisher Scientific, Waltham, MA, USA) was diluted in Tris‐EDTA buffer to obtain the staining solution, of which 100 μL was added per well. Finally, the fluorescence signal emitted at 550 nm was measured using a plate reader (Infinite M Plex, TECAN, Männerdorf, Switzerland). To quantify the concentration of DNA in our samples, we compared the signal intensity to samples in a standard curve with known concentrations.

### 2.7 Live‐Dead‐Assay

At a concentration of 25 mg/mL, FDA (Sigma‐Aldrich, Merck Technology, St. Louis, MO, USA) was dissolved in 99.7% Acetone (Carl‐Roth GmbH, Karlsruhe, Germany).

Following 7 days of treatment, cell culture medium was discarded, the wells washed with PBS and subsequently, 0,4 μL FDA staining solution was added to 1 mL PBS to incubate in the dark (*n* = 3). After 5 min, 20 μL of 1 mg/mL PI (Sigma‐Aldrich, Merck Technology, St. Louis, MO, USA) staining was added to the incubating staining solution and discarded after 5 s. After two further steps of washing, the cells were imaged using an inverted epifluorescence microscope (Zeiss Axio Observer, Zeiss Germany). Standardised recordings and uniform post‐processing procedures were employed to ensure consistency. Six images per well were used to quantify the percentage area of live and dead cells in Fiji/ImageJ (https://imagej.net/ij/9) using the macro “measure stack” as described previously [29].

### 2.8 Enzyme‐Linked Immunosorbent Assay (ELISA)

To measure the protein levels of human cytokines in the cell culture supernatant of each experimental group, the following ELISA kits were used: PDGF‐AB/BB, VEGF and TGF‐β1 (R&D Systems, Bio‐Techne, Minneapolis, MN, USA). Cells were cultured without a complete change of media but fed with 200 μL of fresh medium on day 3. After 7 days, the medium was collected, centrifuged to separate cell debris and aliquoted for ELISAs. Simultaneously, small aliquots of each growth supplement, namely PRFe, PLP, and FBS, were collected on the day of preparation, on day 3 and on day 7. For each day, the supplements were pooled (*n* = 3) and stored at −80°C. Subsequently, we quantified the concentration of cytokines in the growth supplement using ELISA. The human protein levels were determined according to the manufacturer's instructions for each kit and optical density was measured using a plate reader (Infinite M Plex, TECAN, Männerdorf, Switzerland).

### 2.9 Multiplex qPCR

Cells were snap frozen in RNA Later (Qiagen, Hilden, Germany) and stored at −80°C. We used the TaqMan PreAmp Cells‐to‐CT Kit (Invitrogen, Thermo Fisher Scientific, Waltham, MA, USA) and followed the manufacturer's recommended procedures for the preparation of our samples. After thawing, cells were washed in cold PBS and lysed using a 1:100 DNase 1 lysis solution. Following reverse transcription at 37°C for 60 min and 95°C for 5 min, the generated cDNA underwent preamplification (conditions: 95°C for 10 min, afterwards 10 cycles of 95°C for 15 s and 60°C for 4 min). Fluorescence‐labelled probe‐based multiplex RT‐qPCR was performed with 5 μL of each cDNA sample and 15 μL of the TaqMan GenExpression Master Mix in a real‐time thermocycler (qTowerG, Analytik Jena, Germany). We used the Eurofins qPCR‐ASSAY software to design the primer sequences, which are listed in Table 2. The experiment was performed in biological triplicates (*n* = 3). The relative gene expression was normalised to the corresponding Housekeeping Index, consisting of the average of GAPDH. Afterwards, the transcript levels were calculated as 2^−ΔΔCt^, in which ΔΔCt = ΔInduction—Δcontrol (basal medium).

**TABLE 2:** Primer sequences obtained from Eurofins Scientific LE (Luxemburg).

Gene	Name	NCHBI reference sequence	Sequence
GAPDH	Glyceraldehyde‐3‐phosphate dehydrogenase	NM_001256799	Left: ACA TCA TCC CTG CCT CTA C
			Right: CTG CTT CAC CAC CTT CTT G
SOX2	SRY‐box transcription factor 2	NM_003106	Left: GCT CGC AGA CCT ACA TGA AC
			Right: GGA GGA AGA GGT AAC CAC AG
JUN	Jun Proto‐Oncogene, ATP1‐trancription factor subunit	NM_002228	Left: CTG AAA CAG AGC ATG ACC C
			Right: TGC TGG ACT GGA TTA TCA GG
PTGS2/COX2	Prostaglandin‐endoperoxide synthase 2/mitochondrially encoded cytochrome c oxidase II	NM_000963.4	Left: ATC TAC CCT CCT CAA GTC CC
			Right: CGC ATA CTC TGT TGT GTT CC
PXN	Paxillin	NM_001080855	Left: ACT ACC ACA ACC TCT TCT CC
			Right: ACC AAA GAA GGC TCC ACA C
RPS6KA4	Ribosomal protein S6 kinase A4	NM_001006944	Left: ATG TTC ACC CAC CTC TAC C
			Right: AGT CCA GCA GCA CAT TCT C
COL1A1	Collagen type 1 alpha 1 chain	NM_000088	Left: ACG AAG ACA TCC CAC CAA TCA C
			Right: TCA TCG CAC AAC ACC TTG CC
NANOG	Homebox protein NANOG isoform 1	NM_024865.4	Left: TCT CTC CTC TTC CTT CCT CC
			Right: AGT TCT GGT CTT CTG TTT CTT G

### 2.10 Immunocytochemical Staining

Cells were seeded at a density of 2 × 10^4^ per well on cover glasses (Sarstedt AG & Co. KG Nuermbrecht, Germany) in 24‐well plates (Greiner Bio‐one, Frickenhausen, Germany) and treated with growth supplements according to our study design. After 24 h, 72 h and 7 days, the cell culture medium was discarded, and cells were washed with PBS twice. A 4% formaldehyde (Carl Roth, Karlsruhe, Germany) solution was used to fix cells for 20 min at room temperature and cells were then incubated in a blocking buffer containing PBS, 1% BSA and permeabilized by 0.03% Triton X‐100 (Carl Roth, Karlsruhe, Germany) for 30 min. The cells were then washed twice with PBS and then incubated with primary antibodies TGF‐beta1 (Human LAP (TGF‐beta 1) Antibody, R&D Systems, Bio‐Techne, Minneapolis, MN, USA) and CD44 (CD44 Monoclonal Antibody (IM7), Thermo Fisher Scientific, Waltham, MA, USA) for 1 h. Subsequently, the cells were washed again with PBS twice. Following the primary antibody incubation, appropriate secondary antibodies (IgG H&L) (Alexa Fluor 555, Abcam, Cambridge, UK) were added for 1 h.

To visualise the cell nuclei, DAPI (4′‐6′‐diamidino‐2‐phenylindole, Carl Roth, Karlsruhe, Germany) staining was performed by incubating cells for 5 min at room temperature.

### 2.11 Data Analysis

Each experiment was conducted in biological triplicates and technical duplicates. The statistical analysis was performed in GraphPad Prism (V10), with data tested for Gaussian distribution using a Shapiro–Wilk test. If the data followed a Gaussian distribution, statistical analysis was conducted using student's *t*‐test or ANOVA for two or more variables. For significant results, a Bonferroni test was applied as post hoc analysis. Statistical significance was determined to be at least *p* < 0.05.

## 3 Results

### 3.1 ASC Characterisation

Cells demonstrated uniform positive staining for the typical markers of ASCs such as CD13 (99.3% ± 0.9%), CD29 (97% ± 1.5%), CD44 (97.2% ± 2.3%), CD73 (99.4% ± 0.8%), CD90 (99.1.% ± 1.4%) and CD105 (87.8% ± 7.3%), while exhibiting homogeneous negative expression for primary negative markers including CD31 (1.8% ± 2.9%), CD45 (10.7% ± 5.7%) and CD235a (0.4% ± 0.3%). Moreover, these cells exhibited characteristics such as plastic adherence and self‐renewal capability, thus meeting the classification criteria for ASC [26, 30]. Additionally, previous work from our department has elucidated the differentiation potential of the ASC we isolated as part of the characterisation [31].

### 3.2 Cell Viability and Proliferation

**FIGURE 2:**
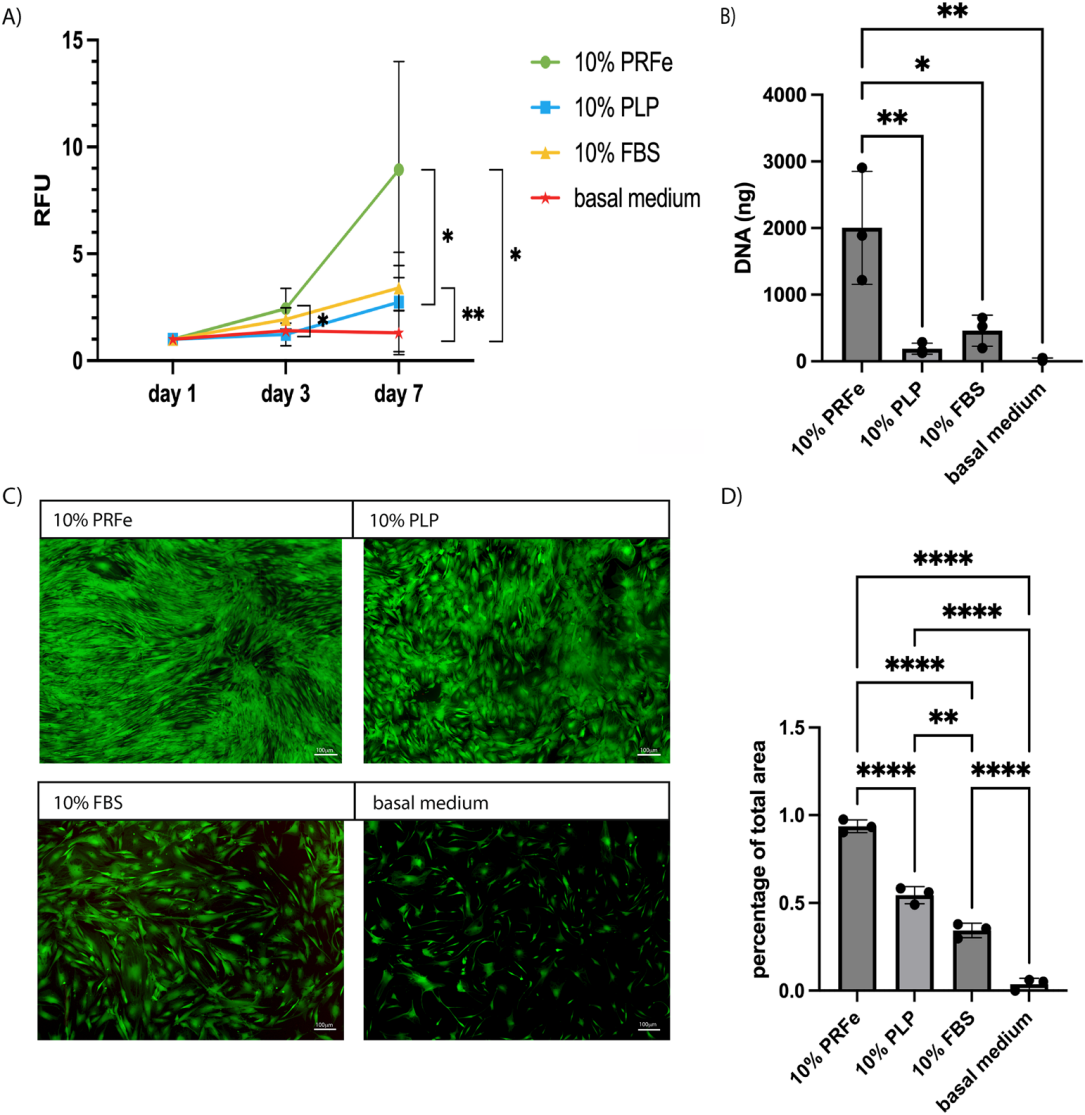
The addition of PRFe increased ASC proliferation significantly: (A) The resazurin based AlamarBlue‐Assay shows constant increase in cell viability measured in relative fluorescence units (RFU) relatively to day 1. (B) After 7 days of incubation the total amount of DNA per well was quantified using a PicoGreen‐Assay. (C) Live‐Dead‐Staining on day 7 using fluorescein diacetate (green) to label vital cells and proprium iodide (red) for dead cells captured at ×5 magnification. (D) growth analysis by estimation of covered well surface with living cells at day 7; *n* = 3; data are shown as means ± standard deviation; *p < 0.05; **p < 0.01; ***p < 0.001, ****p < 0.0001 by two‐way‐ANOVA (A) and one‐way‐ANOVA (B and D).

We studied the impact of three growth supplements on ASC‐vitality in a 2D cell culture over a 7‐day period. Generally, the metabolic activity measured in the AlamarBlue‐Assay increased in all groups over time except for the basal medium only group. Moreover, the addition of PRFe significantly increased the cell viability of ASCs on day 7 when compared to other growth supplements (*p* < 0.05) (Figure 2A).

This finding is supported by the results of the Pico‐Green Assay which showed a significant increase in DNA content in the PRFe group when compared to the other groups (*p* < 0.05 and *p* < 0.01) on day 7 (Figure 2B). Fluorescence staining of unfixed cells to evaluate the fraction of live and dead cells revealed that the PRF group had a higher number of vital cells when compared to the other groups (Figure 2C). Additionally, cell growth was analysed by calculation of covered well surface (Figure 2D). Here, PRFe showed significantly higher (*p* < 0.0001) covered well surface (90%) when compared to all other groups. Pure basal medium was inferior to all other groups.

### 3.3 Gene Expression

**FIGURE 3:**
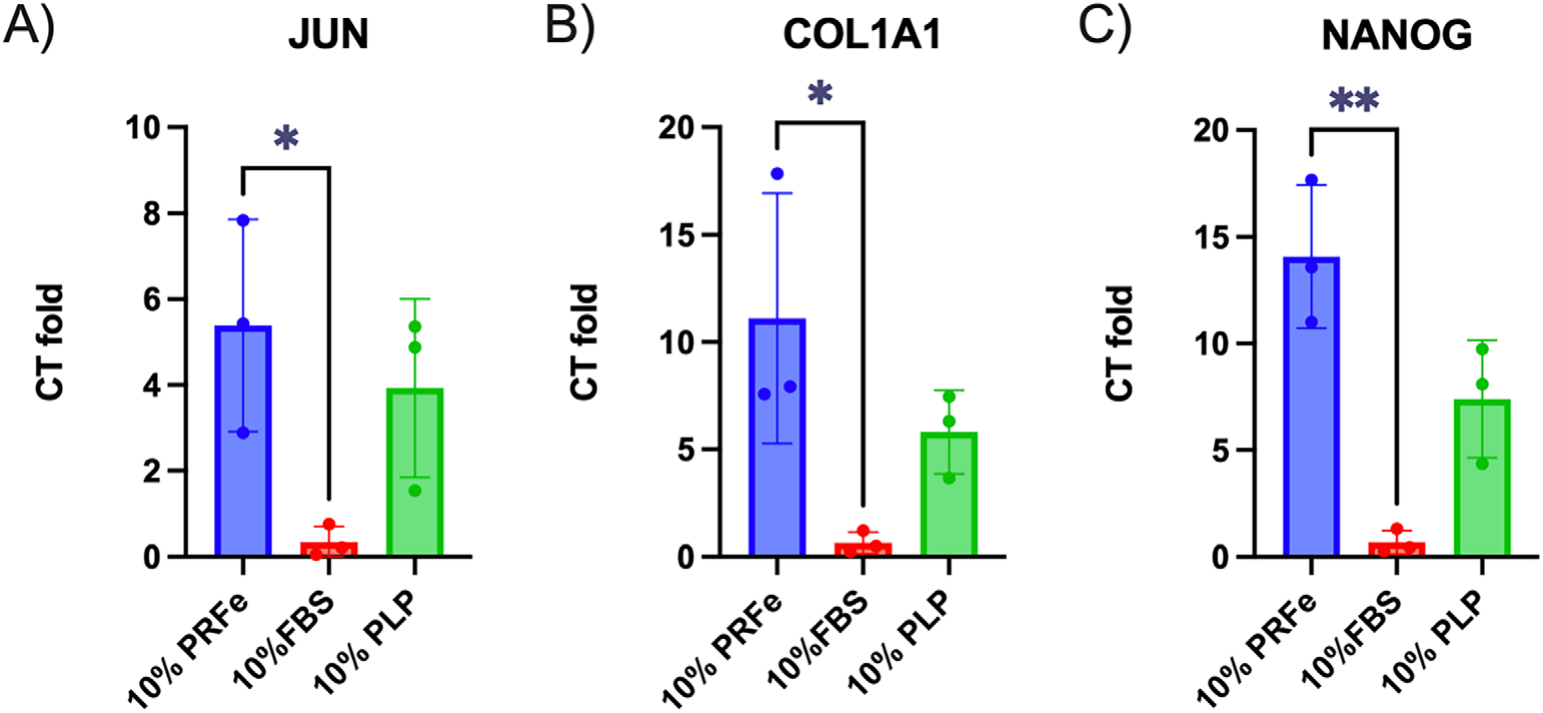
Relative gene expression of ASC in a 2D culture using FBS and PRFe as growth supplement for 7 days analysed by multiplex‐RT‐PCR when compared to basal medium. The following genes were analysed: (A) Jun proto‐oncogene, (B) Collagen type 1 alpha 1 chain, (C) Nanog homebox, (*n* = 3, data are shown as mean value ± SD; *p < 0.05; **p < 0.01 by two‐way‐ANOVA).

Compared to the 10% FBS group, a significant increase in the expression of JUN, COL1A1 (both *p* < 0.05) and NANOG (*p* < 0.01) was observed in the PRFe group after 7 days (Figure 3A–C). Exposure to PLP also resulted in an upregulation of these genes, although the increase was less pronounced and did not reach statistical significance. Likewise, treatment with 10% PRFe also led to an upregulation of SOX2 (twofold), PXN (1.5‐fold), COX2 (twofold) and RPS6KA4 (3.6‐fold) when compared to incubation with 10% FBS. However, due to the high standard deviation, these results were not statistically significant (data not shown).

### 3.4 Growth Factor Secretion

**FIGURE 4:**
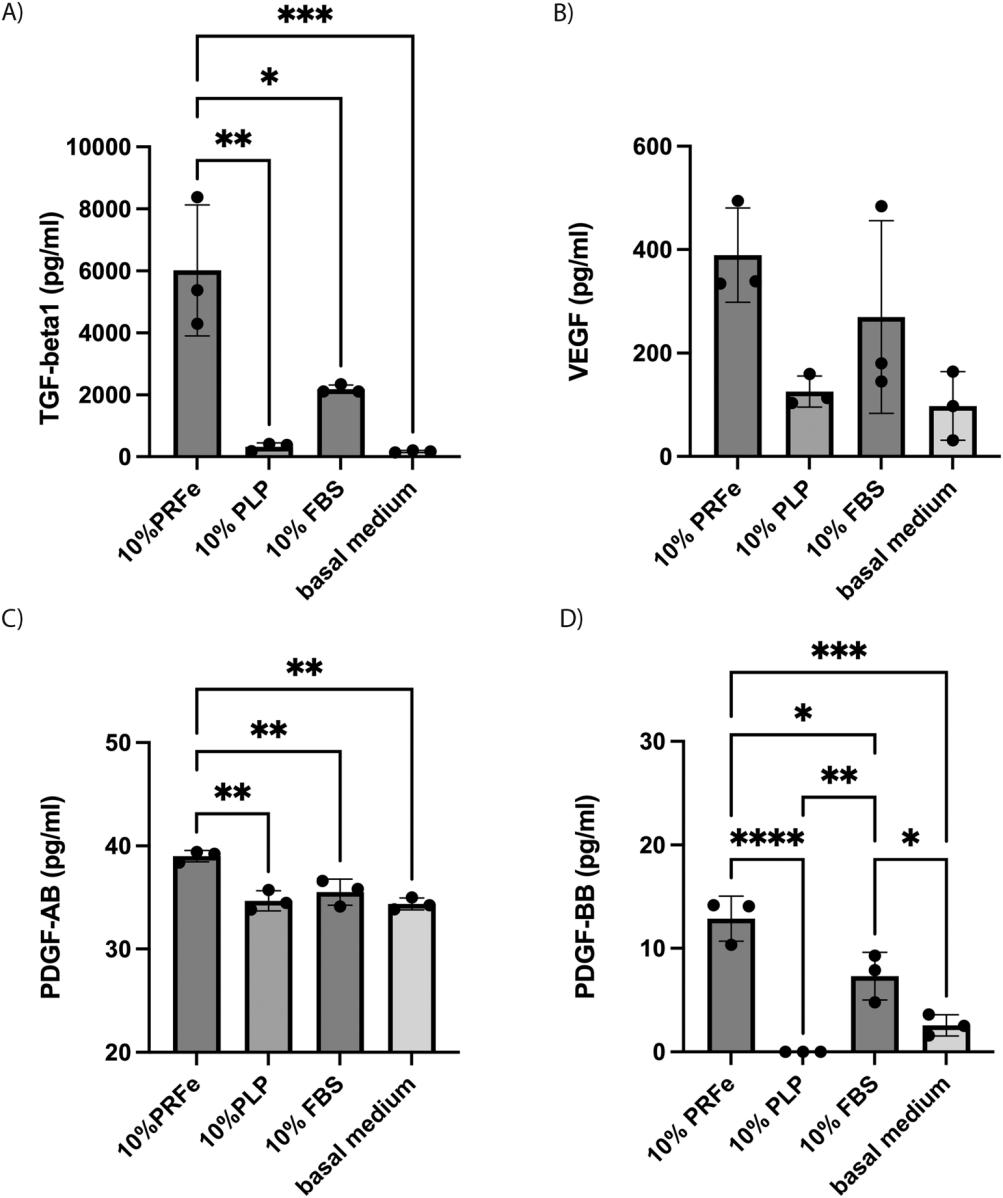
PRFe‐treatment enhances the secretion of cytokines after seven days of cell culture. We investigated the cytokine levels in the cell culture medium of each experimental group (*n* = 3): (A) TGF‐β1, (B) VEGF, (C) PDGF‐AB and (D) PDGF‐BB in cell culture supernatants (*n* = 3, data are shown as mean value ± SD; *p < 0.05; **p < 0.01, ***p < 0.001, ****p < 0.0001 by two‐way‐ANOVA).

**FIGURE 5:**
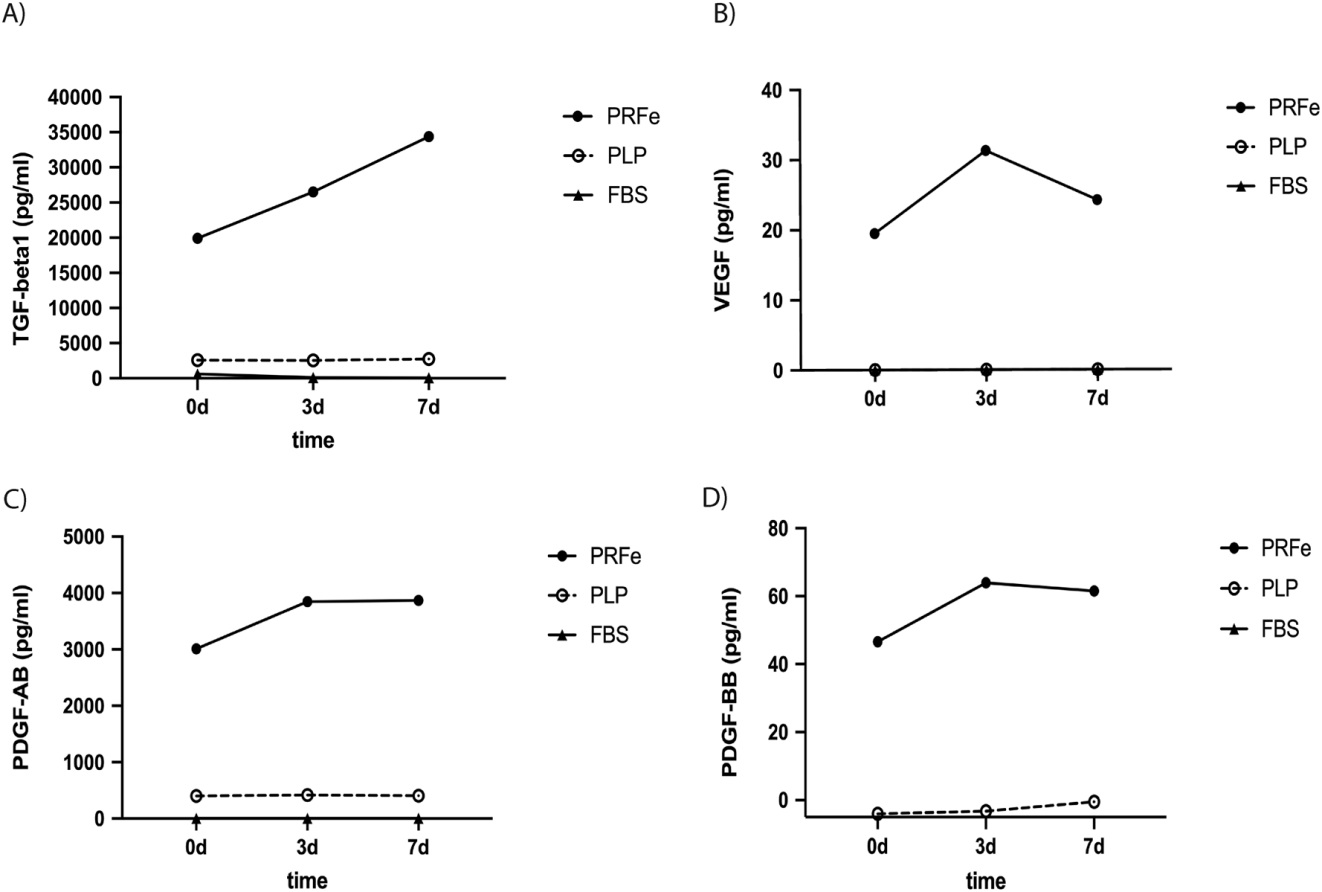
ELISA measurement of cytokines within pooled growth supplements (*n* = 3, i.e., for each data point pooled supplement of three different experiments), (A) TGF‐β1, (B) VEGF, (C) PDGF‐AB and (D) PDGF‐BB within the growth supplements PRFe, PLP and FBS.

To identify cell signalling cascades involved in the observed effects of PRFe on ASC, we measured the concentration of several well‐known cytokines known to be present in PRFe within the cell culture supernatant (Figure 4) as well as in the growth supplements of each corresponding experimental group (Figure 5). These included PDGF‐AB, PDGF‐BB, VEGF and TGF‐β1 [3, 12]. After 7 days of culture, the concentration of TGF‐ß1 was significantly higher (3‐ to 10‐fold increase; *p* of at least < 0.05) in cells treated with PRFe when compared to the other groups (Figure 4A). ELISA of TGF‐β1 in the growth supplements themselves over a period of 7 days indicated that the concentration was the highest in PRFe, even rising over time. PLP maintained a stable concentration of 2500–3000 pg/mL, whereas FBS showed a gradual decrease in TGF‐β1 concentration over time with values under 100 pg/mL (Figure 5A).

VEGF secretion was the highest in the cell culture supernatant of cells treated with 10% PRFe compared to all other groups, although the difference did not reach statistical significance (Figure 4B). Analysis of growth supplements (PRFe, PLP and FBS) demonstrated VEGF to be present only in PRFe, with a peak concentration at day 3 (Figure 5B).

The concentration of PDGF‐AB in the supernatant was elevated in all groups, ranging between 30 and 40 pg/mL, with a significantly higher concentration observed in the PRFe‐group (Figure 4C). When comparing PDGF‐AB levels in the growth supplements, it was found that PRFe had the highest concentration and showed a plateau after 3 days in culture (Figure 5C).

Like PDGF‐AB, the concentration of PDGF‐BB in the cell culture supernatant was significantly higher in cells treated with PRFe when compared to the other groups (*p* of at least < 0.05) (Figure 4D). Moreover, the analysis concentration of PDGF‐BB in the growth supplements was highest in PRFe after three days of culture, with a slight decrease over time (Figure 5D).

### 3.5 Immunocytochemistry

**FIGURE 6:**
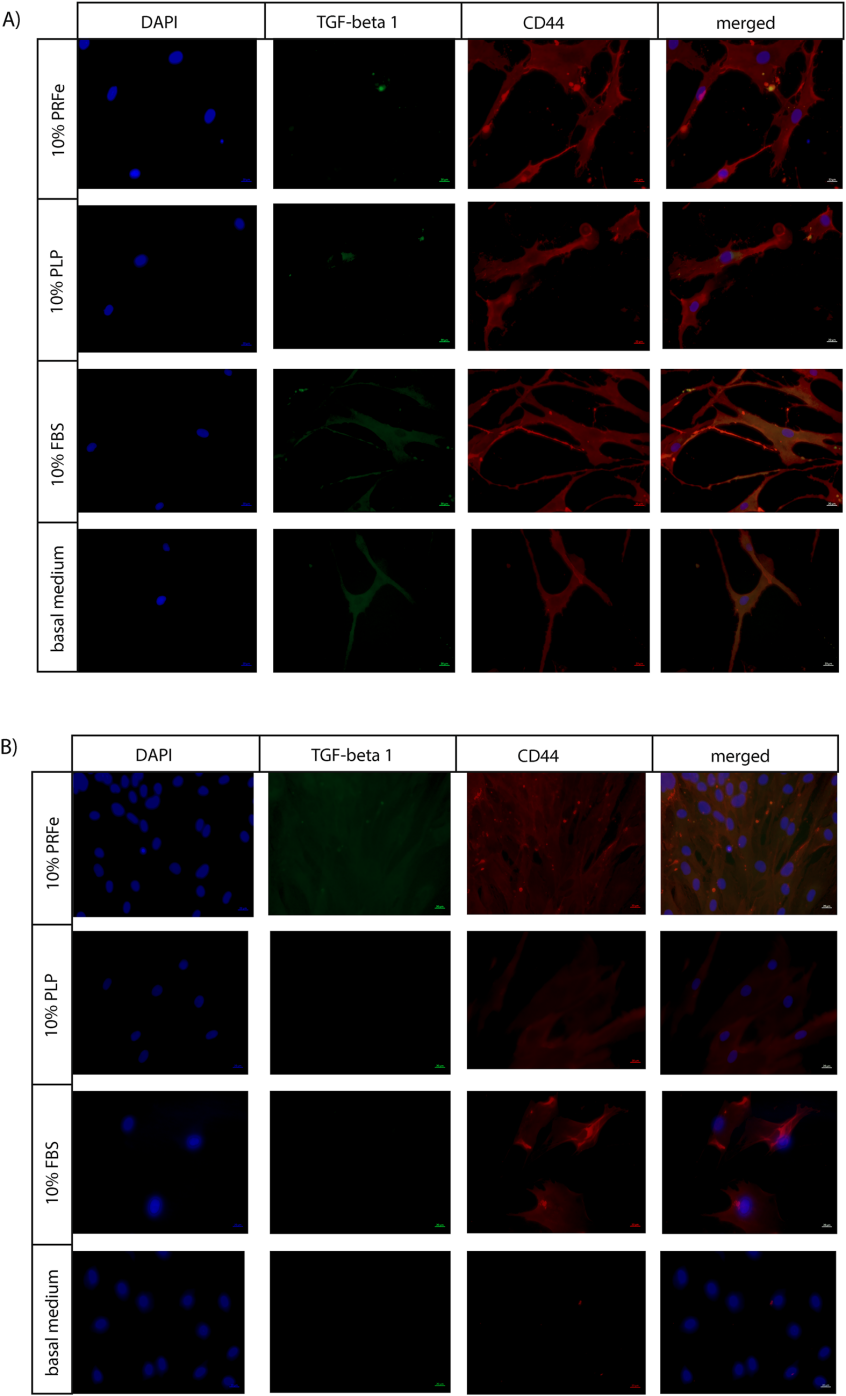
Immunocytochemical staining was performed on ASC treated with 10% PRFe, 10% PLP, 10% FBS or basal medium only after 24 h and 7 days of incubation. The cells expressed CD44, a pluripotency marker and TGF‐β1 at 24 h. However, positivity for TGF‐β1 remained only when the cells were treated with 10% PRFe after 7 days.

After 24 h of incubation, ASC in all experimental groups expressed CD44 surface markers and demonstrated spindle‐like morphology. Staining for intracellular TGF‐beta1 was positive for 10% FBS and basal medium but only partially for 10% PLP and PRFe (Figure 6A).

Conversely, staining after 7 days revealed TGF‐β1 in 10% PRFe. Moreover, these ASC expressed a higher density of CD44 and a more compact/smaller morphology (Figure 6B). As expected, cells exposed to basal medium without an additional growth supplement lost the ability to express CD44 and TGF‐β1.

## 4 Discussion

In this study, we demonstrate a significant effect of PRFe on ASC proliferation and viability in vitro. Indeed, 7 days after incubation, the DNA content per well, cell viability via AlamarBlue‐Assay, as well as the area percentage covered by vital cells was highest in the PRFe group (Figure 2A–D). These findings confirm the results of several other studies [32, 33, 34]. Thus, it was previously demonstrated that PRP and/or PRFe increased cell viability as well as cell number and induced S‐phase in ASC [32, 35]. However, to our knowledge, this study is the first to provide new insights into the differential effects of the two autologous regenerative products, PRFe and PLP, on the biological properties, such as cytokine secretion and gene expression of ASC. Another notable aspect is the comparison with the commonly used growth supplement in cell culture, FBS, which, in summary, rendered more favourable results than PLP but remains inferior to the regenerative impact of PRFe.

It is likely that trophic factors, found in PRFe, induce these effects either by stimulating ASCs directly and/or by inducing the secretion of subsequent cytokines that increase proliferation and cell viability from ASC themselves. In our study, we found significantly elevated levels of TGFβ‐1, VEGF and PDGF‐AA and BB in ASC cultures as well as in PRFe itself (Figures 4 and 5). Indeed, these cytokines have been repeatedly shown to increase proliferation and cell viability in the mesenchymal cell lineage and are highly likely to have played a role in inducing the observed effects on cell viability (Figure 2) [9, 36, 37]. For this, a likely intracellular mechanism is via the modulation of the PKB/Akt and ERK pathways via these and other cytokines found in PRFe and/or PRP, as previously demonstrated by Fukaya et al. and others [38, 39]. Additionally, due to the observed upregulation of Jun proto‐oncogene and high levels of PDGF‐AB in PRFe, we demonstrate that PRFe additionally activates the PDGF Pathway in ASC (Figures 3A and 4C) [40]. Likewise, the elevated expression (1.5 to 3.6‐fold) of COX2, PXN and RPS6KA4 indicates upregulated VEGF and TGF‐ß pathways in ASC incubated with PRFe. However, this remains suggestive as these findings were not significant when compared to controls.

Interestingly, we observed a higher expression of Nanog homeobox genes (Figure 3C) as well as a persistence of CD44 expression in ASC incubated with PRFe after 7 days (Figure 6B). Similar to previous studies, these findings suggest a persistence of pluripotency or stemness due to incubation with PRF (and/or PRP) [34]. Until now, it remains unclear which factors precisely, found in PRF, are predominantly responsible for this effect. Previously, VEGF as well as TGF‐β1 and two have been found to maintain the stemness in mesenchymal stem cells [36]. In the present study, we found increased levels of these cytokines in our cell cultures (Figures 4A,B and 5A,B). Moreover, we demonstrate a significantly elevated expression of TGF‐β1 molecules in ASC incubated with PRFe after 7 days (Figure 6B). Indeed, TGF‐β signalling was found to inhibit adipocyte differentiation of mesenchymal stem cells (MSC) and bone‐marrow stem cells (BMSC) via SMAD3 signalling [41, 42, 43]. Increased levels of TGF‐β1 and 2 as well as VEGF are therefore likely to be responsible for the persistence of stemness we observed in ASC incubated with PRFe. Likewise, elevated levels of COL1A1 expression in cells treated with PRFe are likely to be caused by TGF‐β1 (Figure 3B). This mechanism was demonstrated in various previous studies [44, 45].

Compared to PRF, its precursor PRP requires a longer preparation time and the addition of anticoagulants, which may negatively affect the wound healing and regenerative properties of the platelet concentrate [6, 7]. Moreover, when compared to PRP, PRF allows for a stable secretion of growth factors for up to 7 days, due to the entrapment of cytokines and thrombocytes in fibrin [3, 9, 10, 11]. After the initial conversion of the solid PRF matrix into PRFe, which begins after 30 min and remains stable for at least 7 days, we suspect that only a minimal amount of bioactive factors is lost. We observed this effect for crucial cytokines, namely TGF‐β1 and 2, VEGF and PDGF‐AA and BB (Figure 5). Therefore, it seems likely that a prolonged and stable elevation of these cytokines contributes to the beneficial properties of PRFe in tissue regeneration in vitro, in vivo and in clinical studies [2, 46, 47, 48, 49, 50].

The conversion process takes place rapidly, typically within 30 min, during which the growth factors are released from the fibrin matrix into the surrounding liquid fraction. As a result, PRFe retains its potent regenerative capabilities, which may help maintain the beneficial effects over a longer period, further enhancing tissue repair processes.

Likewise, we demonstrate in the current study that elevated levels of TGF‐1, PDGF‐AA/BB and potentially VEGF in PRFe are likely to have caused ASC proliferation and regenerative potential (Figures 2 and 3). Thus, combining PRFe and ASCs would potentially help to solve a critical problem often found with in vivo use of ASCs, as it could create an environment that allows for a significantly higher number of cells to survive [51, 52]. This may especially hold true for less preferable environments, such as chronic wounds.

The results of the present study provide interesting insights into the possible beneficial effects of PRFe on ASC. However, a more detailed investigation into which specific growth factors in PRFe are responsible for the effects observed by us and others is warranted and should therefore be the subject of future investigations.

For the PRFe group, we used 10% PRFe to supplement cell cultures as this concentration allows a better comparison to cell cultures with common growth medium (+10% FBS). Importantly, an analysis of a possible dose‐dependent effect of PRFe on ASC is missing in the current study. Knowledge of the optimum concentration would be an important baseline for follow‐up experiments. This seems particularly relevant for in vivo experiments that would allow for a better proof of concept of the in vitro findings presented in the current study: while the beneficial effects of adding PRFe to ASCs seem biologically plausible, we can only hypothesise whether this effect would translate to an in vivo setting. We therefore encourage future animal studies to provide foundational data for clinical translation of our findings.

Another weakness in the current study is the relatively small sample size. This seems particularly relevant with regard to the expression of SOX, PXN, COX2 and RPS6KA4 genes in ASC incubated with PRF (data not shown). Here, we observed an elevated gene expression between 1.5 to 3.6‐fold. Equally, we see similar limitations for the measurements of VEGF in cell culture supernatant (Figure 4B). In our experiments, we observed elevated concentrations of VEGF in cultures with PRFe. However, similar to the findings on the expression of the above‐mentioned genes, the small sample size (*n* = 3) and a relatively large standard deviation, our statistical analysis resulted in no significance for these effects. It therefore seems likely that we would have observed significant changes with a larger sample size, similar to previous studies [46, 53, 54].

Nevertheless, this study demonstrates the beneficial effects of using PRFe as a possible adjunct for the cultivation of ASC, superior to the other growth supplements examined in the experimental setup. Moreover, we show that this effect is likely to be mediated by elevated TGF‐β1, VEGF and PDGF‐AA and BB. Notably, we emphasise the pivotal role of TGF‐β1, given its association with the upregulation of collagen 1A1, a critical component of extracellular matrix remodelling and tissue repair. Therefore, we see an interesting possibility of combining PRF and ASC for the application in vivo as well as in the clinical setting for future studies.

## Ethics Statement

The study was conducted according to the guidelines of the Declaration of Helsinki and approved by the local ethics committee (registration number: 21‐0138).

## Consent

Informed consent was obtained from all individual participants included in the study.

## Conflicts of Interest

The authors declare no conflicts of interest.

## Data Availability

Data available on request from the authors.
